# DPNet: Scene text detection based on dual perspective CNN-transformer

**DOI:** 10.1371/journal.pone.0309286

**Published:** 2024-10-21

**Authors:** Yuan Li

**Affiliations:** School of Physics and Electronic-Electrical Engineering, ABA Teachers University, Wenchuan, Aba Tibetan and Qiang Autonomous Prefecture, Sichuan, China; Dalian Maritime University, CHINA

## Abstract

With the continuous advancement of deep learning, research in scene text detection has evolved significantly. However, complex backgrounds and various text forms complicate the task of detecting text from images. CNN is a deep learning algorithm that automatically extracts features through convolution operation. In the task of scene text detection, it can capture local text features in images, but it lacks global attributes. In recent years, inspired by the application of transformers in the field of computer vision, it can capture the global information of images and describe them intuitively. Therefore, this paper proposes scene text detection based on dual perspective CNN-transformer. The channel enhanced self-attention module (CESAM) and spatial enhanced self-attention module (SESAM) proposed in this paper are integrated into the traditional ResNet backbone network. This integration effectively facilitates the learning of global contextual information and positional relationships of text, thereby alleviating the challenge of detecting small target text. Furthermore, this paper introduces a feature decoder designed to refine the effective text information within the feature map and enhance the perception of detailed information. Experiments show that the method proposed in this paper significantly improves the robustness of the model for different types of text detection. Compared to the baseline, it achieves performance improvements of 2.51% (83.81 vs. 81.3) on the Total-Text dataset, 1.87% (86.07 vs. 84.2) on the ICDAR 2015 dataset, and 3.63% (86.72 vs. 83.09) on the MSRA-TD500 dataset, while also demonstrating better visual effects.

## Introduction

Scene text detection has emerged as an active area of research with extensive practical applications in fields such as scene understanding, autonomous driving, navigation, localization, and photo translation. Currently, methods for deep learning-based scene text detection can be classified into two primary types: regression-based and segmentation-based methods. Although these two types of algorithms offer significant advantages over traditional algorithms that rely on hand-crafted features, they are also plagued by several issues. The regression algorithm, which is based on candidate boxes, involves operations such as setting a preselection box and removing duplicates before producing the final prediction box. In contrast, the effectiveness of the prediction box generated by segmentation-based algorithms heavily depends on the precision of the segmentation process. As research progresses, it has been discovered that integrating the strengths of CNNs and transformers [1] within the same network can significantly enhance the model’s detection performance, addressing the issues associated with the two aforementioned methods.

Although the field of scene text detection has been developing for many years and has achieved significant progress, it continues to face numerous challenges. The main reasons are as follows: the text itself varies in font sizes, adopts arbitrary and variable shapes, appears in random positions, and has indeterminate directions; the scene images may exhibit issues such as occlusion, distortion, bending, color distortion, and complex backgrounds. The original Differentiable Binarization (DBNet) [2] algorithm employs a differentiable binarization technique to streamline the post-processing steps and addresses the issue of non-differentiability of gradients during training. However, the network does not fully utilize high-level semantic and spatial location information. The lack of sufficient global context and spatial information leads to false detections or missed detections, thus limiting the network’s positioning capabilities. Addressing the aforementioned challenges, this paper, inspired by the Vision Transformer (ViT) [3], proposes an effective scene text detection network based on a dual-perspective CNN-transformer architecture. On the one hand, on the basis of the traditional ResNet, this paper proposes a channel enhanced self-attention module (CESAM) and a spatial enhanced self-attention module (SESAM), which perceive the text information in terms of channel and space, and extract the text vision features more robustly. On the other hand, the feature decoder proposed in this paper is mainly realized by using the traditional convolution layer, which comprehensively takes into account the vision features and semantic information of the text during the detection process, and promotes finer feature extraction, thus improving the detection performance of arbitrary text.

In summary, the principal contributions of this paper are as follows:

1. CESAM and SESAM are proposed to extract text attention from each channel and spatial location. They can perceive global information within the image, not just local features, which helps to improve the performance of text detection in images;
2. An efficient and novel feature decoder is proposed, integrating multi-scale text to fully combine global and local feature information across different scales;
3. A large number of experiments confirm that the network is effective and can easily adapt to various scene text detection datasets with minimal adjustments to the architecture.

The rest of this paper is organized as follows. The ‘Related Work’ introduces the related work on the algorithm of this paper. The ‘Method’ provides a detailed description of the proposed method, including the model architecture and loss function. The ‘Experiments and Results’ describes the experimental setup, including the datasets and running environment, compares the proposed method with other algorithms both objectively and subjectively, and discusses the experimental results. Finally, the ‘Conclusions’ summarizes the main contributions of this paper and further analyzes potential directions for future research development.

## Related work

CNNs: CNNs have been widely studied in the field of scene text detection. Significant improvements in representation are achieved through methods that involve making networks deeper [4], wider [5, 6], increasing cardinality [7], and dynamically enhancing features [8]. Multi-level feature fusion, which explicitly incorporates shallow low-level features into the output layer, reduces the model’s focus on these low-level details. This approach enables better capture of high-level semantics and can further enhance the representation ability of CNNs. This approach is ubiquitous across many network architectures and has been extensively studied, as documented in references [9–11]. For instance, within the InceptionNet family [12, 13], outputs from filters of various sizes at the same level are fused to effectively address significant variations in target size.

Attention mechanisms: Neural network architectures for computer vision tasks [14–17] incorporate an attentional mechanism to capture text feature information in the channel, thereby complementing the feature information in the convolutional layer. Li et al. [16] adjusted the receptive fields of their network branches by employing an attention mechanism. SENet [8] incorporates an attention-like module that focuses on the channel relationships between features at each layer. The attention mechanism can learn the correlations among different parts of the input sequence, enabling the model to better understand and process the input information.

Self-attention mechanism and the integration of CNNs: To obtain attention weights for channels or spatial configurations, some studies have integrated the self-attention mechanism with CNNs to guide segmentation. Building on the concept of modeling the interdependence of semantic information, dual attention networks incorporate two types of attentional modules: spatial and channel-based [18]. Attention U-Net [19] introduces an attention gate that suppresses irrelevant regions and accentuates salient features to effectively guide the segmentation task. Although the aforementioned methods enhance performance, further improvement is necessary to advance the extraction of semantic information.

Transformers: Recently, transformers have been widely used in natural language processing tasks, delivering state-of-the-art performance in various benchmark tests [20, 21]. It dominates applications in the field such as information extraction, machine translation, and sentiment analysis. The transformer encoder is integrated into the backbone network’s attention operation, facilitating the learning of long-distance dependencies. Many researchers are exploring the application of transformers in various fields, such as adapting transformer architecture for image pixels [22, 23]. Child et al. [23] observed that many of the earlier layers in the network exhibit local connection patterns akin to those in convolution, demonstrating that a hybrid architecture combining transformers and convolution networks is a compelling choice.

Mixed text-image embeddings already utilize transformers with detection bounding boxes as input [24], indicating that most image processing occurs within the convolutional domain. Recently, the Vision Transformer (ViT) [3] architecture, which adapts transformers for computer vision tasks, has been proposed. The self-attention mechanism, implemented using a transformer on the input image, achieves state-of-the-art performance in image classification. As research progresses, the transformer is increasingly being applied to semantic segmentation. Therefore, several ViT variants have been proposed, including DeiT [25], SETR [26], Swin Transformer [27], Swin-UNet [28], TransUNet [29], and LeViT [30]. The hybrid transformer and convolutional model, LeViT, achieves fast inference in image classification with an improved balance of accuracy and efficiency. However, a drawback of the architecture is that it does not fully exploit the feature map information produced by the transformer and convolution layers, particularly for image segmentation. Based on these considerations, LeViT-UNet was proposed [31]. The encoder portion of LeViT-UNet is derived from the LeViT transformer architecture. In the LeViT transformer, the feature maps are upsampled, cascaded, and skip-connected before reaching the decoder. The decoder of LeViT-UNet is constructed using standard convolutional layers. This design effectively integrates the global feature extraction capabilities of the transformer with the local feature representation of CNNs. Bello [32] proposes an approximate attention mechanism that combines content attention with a component of positional attention. In more recent studies, this approach has been explored for various tasks [33, 34]. The Pyramid Vision Transformer (PVT) [35], primarily inspired by ResNet, is employed for image classification. It is primarily employed for instance segmentation tasks. Yuan et al. [36] explore how architectural decisions within CNNs [37] can enhance the performance and efficiency of image processing.

Segmentation-based methods primarily draw from semantic segmentation techniques, considering all pixels within text bounding boxes as positive samples. These methods describe text regions through various representations and then reconstruct text instances via specific post-processing. Zhou et al. introduced an efficient and accurate scene text detector, EAST [38], which predicts text using a convolutional neural network and employs non-maximum suppression to remove redundant text boxes. However, it still falls short in detecting some elongated texts. Long et al. proposed an algorithm, TextSnake [39], which detects arbitrarily shaped scene text via text centerline detection, uses a Fully Convolutional Network (FCN) for prediction, and employs a Disjoint Set and Striding algorithm to output the final text boxes, though its post-processing is relatively complex. Wang et al. introduced a Progressive Scale Expansion Network (PSENet) [40] that segments pixels into text and non-text and uses a scale expansion algorithm to aggregate text pixels, allowing for the detection of text in any shape. However, this model is complex and challenging to train. Wang et al. [41] improved the PSENet framework with the Pixel Aggregation Network (PAN), capable of detecting text of any shape, though it is time-consuming. Baek Y et al. [42] proposed a Character Region Awareness for Text Detection (CRAFT) algorithm based on character probability prediction. This algorithm, trained under weak supervision, captures individual character scores and the connectivity between adjacent characters, suitable for detecting text with consistent spacing but struggles with variable spacing. DBNet [2] introduced a Differentiable Binarization module, which binarizes through an adaptive threshold map, and the threshold map can compute loss, enhancing text detection results and simplifying the post-processing. Compared to other text detection models, DBNet shows significant advantages in effectiveness and performance, making it a commonly used text detection algorithm. However, due to the limited receptive field of convolutional layers in CNNs, which cannot model global semantic information effectively or learn more advanced feature information, there are shortcomings in text feature extraction capabilities and receptive field limitations. To address these issues and effectively reduce false positives and misses, this paper proposes a dual-view CNN-transformer based scene text detection algorithm, DPNet, for handling complex scale variations in scene text. This model focuses on expanding the receptive field through a dual-view CNN-transformer, integrating global and local feature information to localize text regions, thereby learning more detailed text position information and enhancing the capability to acquire contextual feature information. The findings of this paper may serve as a benchmark for comparisons in the efficient application of transformers to scene text detection.

## Method

### The architecture of DPNet

Images from most natural scenes originate from real life, presenting considerable challenges in scene text detection. On one hand, there is diversity in font types, languages, and morphologies, including varied textural structures, multiple colors, and diverse text arrangements. On the other hand, factors such as shooting angles, lighting, occlusions, focusing inaccuracies, and color distortions also pose significant difficulties. The primary challenge lies in the lack of sufficient high-level semantic information extraction and storage capabilities in networks constructed from standard convolutional layers, leading to incomplete feature extraction. Additionally, under complex background conditions, the limited size of the receptive fields in CNNs prevents effective utilization of image features, thus hindering accurate localization of texts of arbitrary shapes. Therefore, this paper introduces CESAM and SESAM based on the CNN-transformer framework from a dual perspective to expand the receptive field, and employs a feature decoder to enrich feature extraction.

The network proposed in this paper (DPNet) and the baseline (DBNet) both consist of three components: Backbone, Neck, and Head, as indicated in Table 1. In DBNet, the Backbone includes ResNet18 and ResNet50, which are responsible for extracting text-related features from images for subsequent network decoding and text localization. Without modifying the fundamental architecture and ensuring the preservation of the network’s original feature information, the CESAM and SESAM introduced in this paper refine the feature expression process of the main network, ResNet18 (ResNet50), from the channel and spatial perspectives, respectively, thereby enhancing the ability to extract text features. The Neck, situated between the Backbone and the Head, is a novel feature decoder designed on the basis of the FPN. It enhances the network’s capability to handle variations in text scale by augmenting the information in different levels of feature maps. The Head is the detection component, responsible for localizing text areas from the output of the feature extraction network. In this study, the Head remains consistent with the baseline, employing Differentiable Binarization. It utilizes the output of the Feature Decoder as the input to the DB module, leveraging previously extracted features to predict text boxes. The final results are then outputted after processing by the DB module.

**Table 1:** Components of the DBNet and DPNet models.

Model	DBNet	DPNet
Module		
Backbone	ResNet	Feature Encoder (ResNet+CESAM+SESAM)
Neck	FPN	Feature Decoder
Head	Differentiable Binarization	Differentiable Binarization

**Fig 1:**
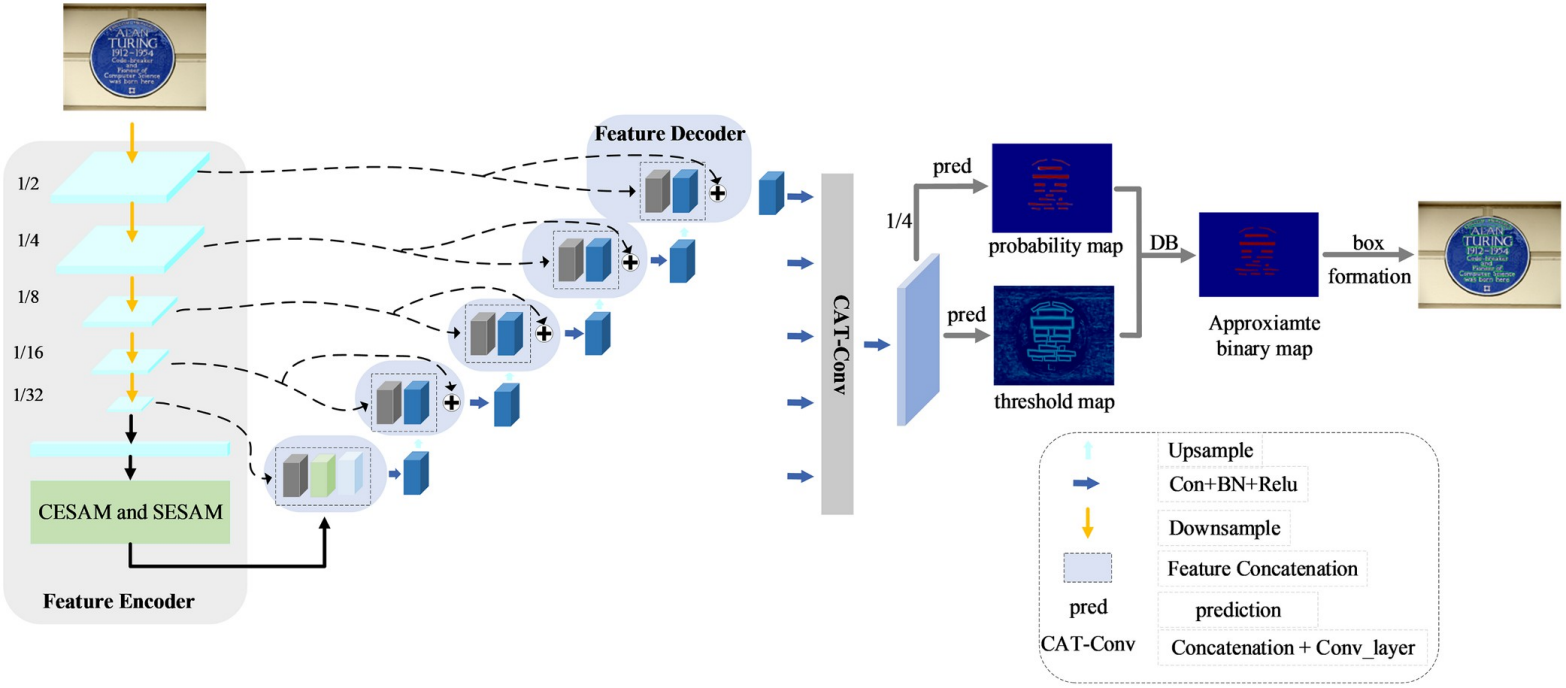
The overall architecture of the proposed DPNet. Firstly, the feature encoder serves as the backbone network of the model, comprising ResNet and the proposed CESAM and SESAM to increase the receptive field. Secondly, the output of the backbone network is processed through the feature decoder to extract more detailed feature information. Finally, the output of the feature decoder is processed through the DB module to produce the final detection result.

Due to the limited receptive field of convolutional layers in convolutional neural networks, which is insufficient for effectively modeling global semantic information, this paper builds upon the DBNet framework to propose a dual perspective CNN-transformer algorithm for scene text detection. The overall framework of DPNet is shown in Fig 1. The entire network architecture comprises three components: a feature encoder, a feature decoder, and a differentiable binarization module. In the part of feature extraction, this paper proposes CESAM and SESAM. By using the feature aggregation ability of these two modules, the features can be optimized from the channel and spatial position respectively, and a more accurate detection box can be obtained. Meanwhile, this paper proposes a feature decoder, which further enriches the text feature information and is relatively efficient without any post-processing module. Overall, these two stages promote each other and together improve the final detection performance.

In general, the CESAM and SESAM introduced in this paper are integrated into the original ResNet backbone network and processed through the feature decoder also proposed in this paper. The purposes of this are as follows:

- Firstly, by integrating CESAM and SESAM, the output feature map is optimized, thereby enhancing the detail and precision of global feature extraction.
- Secondly, the features transmitted by the unique feature decoder produce more accurate and superior feature maps compared to those generated by the original FPN architecture, thereby facilitating the development of a robust system capable of extracting features from multiple perspectives.

Thanks to the architectural design presented in this paper, each pixel in the image not only has information about its immediate neighborhood but also interacts with channel information, thereby minimizing the interference of irrelevant regions in the global information. Simultaneously, this approach effectively expands the perceptual field of each spatial location, allowing for a heightened focus on more contextual information. Taking ResNet-18 as an example, the input image size is represented as (*B*, 3, 640, 640), where ‘B’ denotes the batch size, ‘3’ represents the number of channels, and ‘640×640’ corresponds to the dimensions H×W. Table 2 illustrates the changes in dimensions between the baseline and the method proposed in this paper.

**Table 2:** Changes of channel dimensions of DBNet and DPNet.

	DBNet			DPNet			
block	layer	Input size	Output size	block	layer	Input size	Output size
ResNet	Resblock1	(*B*, 3,640,640)	(*B*, 32,320,320)	ResNet	Resblock1	(*B*, 3,640,640)	(*B*, 32,320,320)
	Resblock2	(*B*, 32,320,320)	(*B*, 64,160,160)		Resblock2	(*B*, 32,320,320)	(*B*, 64,160,160)
	Resblock3	(*B*, 64,160,160)	(*B*, 128,80,80)		Resblock3	(*B*, 64,160,160)	(*B*, 128,80,80)
	Resblock4	(*B*, 128,80,80)	(*B*, 256,40,40)		Resblock4	(*B*, 128,80,80)	(*B*, 256,40,40)
	Resblock5	(*B*, 256,40,40)	(*B*, 512,20,20)		Resblock5	(*B*, 256,40,40)	(*B*, 512,20,20)
	Adjust all output channels to 256 via Conv				Adjust all output channels to 128 via Conv		
			*C*_2_: (*B*, 256,160,160)				*C*_2_: (*B*, 128,160,160)
			*C*_3_: (*B*, 256,80,80)				*C*_3_: (*B*, 128,80,80)
			*C*_4_: (*B*, 256,40,40)				*C*_4_: (*B*, 128,40,40)
			*C*_5_: (*B*, 256,20,20)				*C*_5_: (*B*, 128,20,20)
				CESAM	Transpose Flatten	(*B*, 128,20,20)	(*B*, 400,128)
					Linear Split	(*B*, 400,128)	*q*: (*B*, 4,400,16)
							*k*: (*B*, 4,400,16)
							*v*: (*B*, 4,400,32)
					Self-atten Reshape		(*B*, 128,20,20)
				SESAM	Transpose Flatten	(*B*, 128,20,20)	(*B*, 400,128)
					Linear Split	(*B*, 400,128)	*q*: (*B*, 4,16,400)
							*k*: (*B*, 4,16,400)
							*v*: (*B*, 4,32,400)
					Self-atten Reshape		(*B*, 128,20,20)
FPN			(*B*, 64,160,160)	Feature Decoder	Up Conv		(*B*, 64,160,160)

### Feature encoder

**Fig 2:**
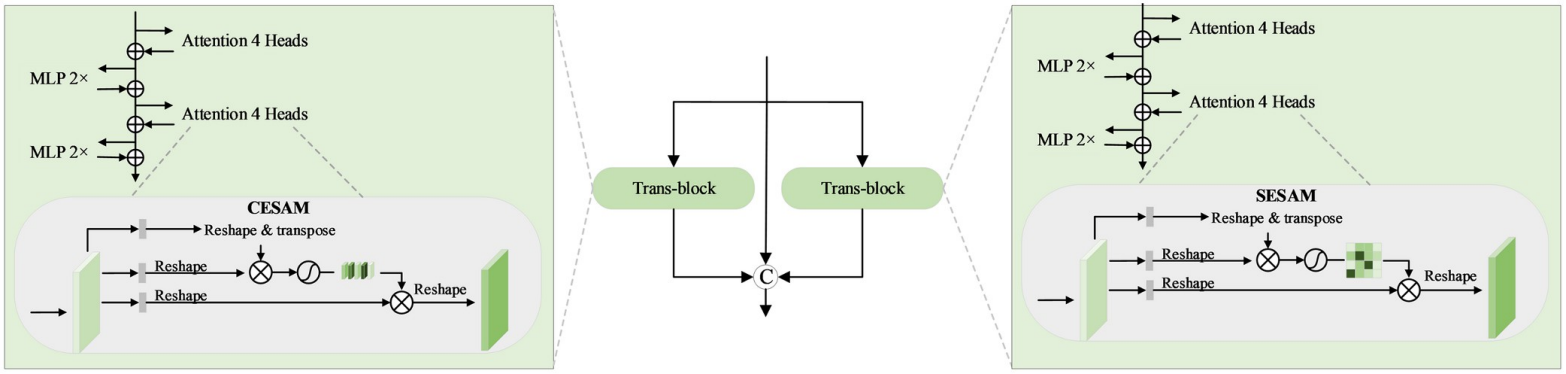
The structural design of the dual perspective transformer high-level semantic feature extraction module. On the left is the design of the Channel Self-Attention Module (CESAM), and on the right is the design of the Spatial Enhanced Self-Attention Module (SESAM). This paper embeds CESAM and SESAM into the original backbone network ResNet to enhance the network’s feature extraction capabilities based on dual perspective.

The feature encoder is a feature extraction module that integrates both channel and spatial dimensions, enabling feature extraction from two distinct perspectives to enhance the precision and granularity of the features obtained. To efficiently gather contextual information from channel and spatial dimensions, this paper introduces CESAM and SESAM, as depicted in Fig 2. These modules are designed to address the relationships within and between text instances. These two modules process queries through multiple layers and then explore the relationships across instances. This approach guides the model in distinguishing between background and text within the image, both in channel and spatial dimensions. The feature encoder extracts specific text features, enhancing the model’s ability to detect text more effectively. The main motivation of this encoder has two aspects: (i) Synthetically learn low-level local features and high-level contextual features. (ii) Obtaining rich text-specific features for subsequent detection. For (i), both local and global context information are crucial for the model to accurately estimate fuzzy details. For (ii), highly representative features that contain rich text-specific information are crucial for the detection process.

The CESAM proposed in this paper demonstrates strong channel feature extraction capabilities, with the output feature of each channel being a weighted sum of all channels’ original features. Similarly, the SESAM introduced in this paper performs a weighted summation of all positional features. The module effectively extracts meaningful spatial features by capturing the spatial dependencies between any two locations on the feature map, thereby updating specific features. By combining the powerful location feature extraction capabilities of SESAM with the channel feature extraction advantages of CESAM, this approach excels in both location and channel feature extraction. Compared to single feature extraction, multi-angle feature extraction provides more accurate and detailed features, enhancing the model’s expressiveness and learning capacity, thereby improving text detection performance. In addition, the two modules introduced not only enhance the robustness of the model but also effectively reduce its sensitivity to overfitting, thereby improving the overall performance and generalization capabilities of the model. Therefore, the hybrid text detection network proposed in this paper possesses an excellent global feature extraction capability that traditional CNNs alone cannot achieve.

Taking ResNet-18 as an example, the output feature map is sequentially obtained as {*C*_2_, *C*_3_, *C*_4_, *C*_5_}. Their sizes are $\left\{\frac{1}{4},\frac{1}{8},\frac{1}{16},\frac{1}{32}\right\}$ of the original size. Because the feature map *C*_1_ is excessively large, occupying half the size of the original image and consuming significant memory, it is discarded. The multi-scale features {*C*_2_, *C*_3_, *C*_4_, *C*_5_} are processed through a conventional convolution layer. Unlike DBNet, which has a channel dimension of 256, this paper standardizes the channel dimension to 128 to reduce the number of parameters and the network’s complexity. From the above analysis, it can be concluded that the lower feature layers (e.g., feature map *C*_2_) contain more texture detail information, while the higher level feature maps (e.g., feature map *C*_5_) encapsulate more semantic information, which is crucial for efficiently and effectively learning global text information.

Therefore, to capture more representative text semantic information, the feature map *C*_5_, which contains features highly correlated with the foreground text, is fed into CESAM and SESAM. These modules predict textual features in parallel while balancing the parameters and complexity of the network. The text channel and spatial perception process actually involves concatenating two sets of perceptual weights with the text features to obtain the fused features. This is achieved by concatenating the output features from CESAM and SESAM with the feature map *C*_5_. The output features acquire attentional information on channels and spatial locations, thereby enriching the feature encoder with both local and global attribute features. Local features are crucial for detecting small and detailed texts in images, while global features are essential for understanding the overall structure of images. By combining local and global features, the feature encoder can accurately obtain text feature information. Consistent with reference [31]. The attentional coding layer is computed as depicted in Eq (1).

$$X\left(q,k,v\right)=\text{softmax}\left(\frac{qk^{\text{T}}}{\sqrt{d_{k}}}+\text{attention}\;\text{bias}\right)v$$ (1)

Where *q*, *k*, *v* are input features, representing the query vector, key vector, and value vector, respectively, and *d*_*k*_ is the dimension of the feature.

Taking CESAM as an example, the specific steps for encoding are as follows:

First step. Three vectors are computed for each input vector to the encoder: a query vector, a key vector, and a value vector. Multiply the input vector by the three weight matrices, which are trained during the model training phase. It is not necessary for these three vectors to be smaller than the dimensions of the encoder’s inputs and outputs, this practice is primarily implemented to stabilize the computation of multiple attention mechanisms. In Table 2, the dimensions of the three vectors for CESAM are given as *q* (*B*, 4, 400, 16), *k* (*B*, 4, 400, 16), and *v* (*B*, 4, 400, 32). In contrast, SESAM features dimensions *q* (*B*, 4, 32, 400), *k* (*B*, 4, 32, 400), and *v* (*B*, 4, 32, 400) for the respective vectors. In this context, ‘4’ represents the number of attention heads. When generating *q*, *k*, *v*, each is divided into four parts. Each part is processed individually, and then the parts are concatenated to form the final vectors. This approach facilitates parameter segregation and enables associated features to be clustered together, simplifying the training process.
Second step. Calculate the attention score as follows: Suppose that the self-attention of the first text in the input is now been computed, this self-attention is then used to score each text within the input. This score determines the level of attention allocated to text at other positions when encoding text at a given position. This score is calculated as the dot product of the query and key vectors. Here, when calculating the attention score for the first position, the query corresponding to the first text is multiplied in turn by the other keys.
Third step. Divide the calculated attention score by the square root of *d*_*k*_.
Fourth step. Adding attention bias to attention maps [32] ensures that the feature map in each dimension retains the original positional information, enabling positional encoding.
Fifth step. The results are normalized using softmax calculations. The attention score after softmax reflects the level of attention that other texts receive during the calculation for the current position. Clearly, the text at the current position typically receives a high score; however, occasionally, other texts related to the current text are also noticed.
Sixth step. Each value vector is multiplied by the corresponding attention score. This process aims to retain the values of the text the paper focuses on, while discarding irrelevant words, to output the attention results.

Within the feature encoder, CESAM and SESAM leverage attention bias to integrate positional information and use multi-head self-attention layers to learn both local and global dependencies within the image. This approach differentiates between background and text in images by examining channel and spatial dimensions. For the same image, different observers may notice and focus on different aspects. The multi-head attention mechanism in the model aggregates these differences, facilitating the comprehensive learning of features from various perspectives, levels, and dimensions, thereby achieving a fusion of local and global information. By utilizing the proposed CESAM, SESAM, and feature decoder, the algorithm aims to enhance the overall network performance.

### Feature decoder

Considering the baseline’s limited focus on local detail information, this paper introduces a feature decoder designed to further enhance the detail of text features, obtain more precise text boxes, and improve the model’s feature learning capability.

**Fig 3:**
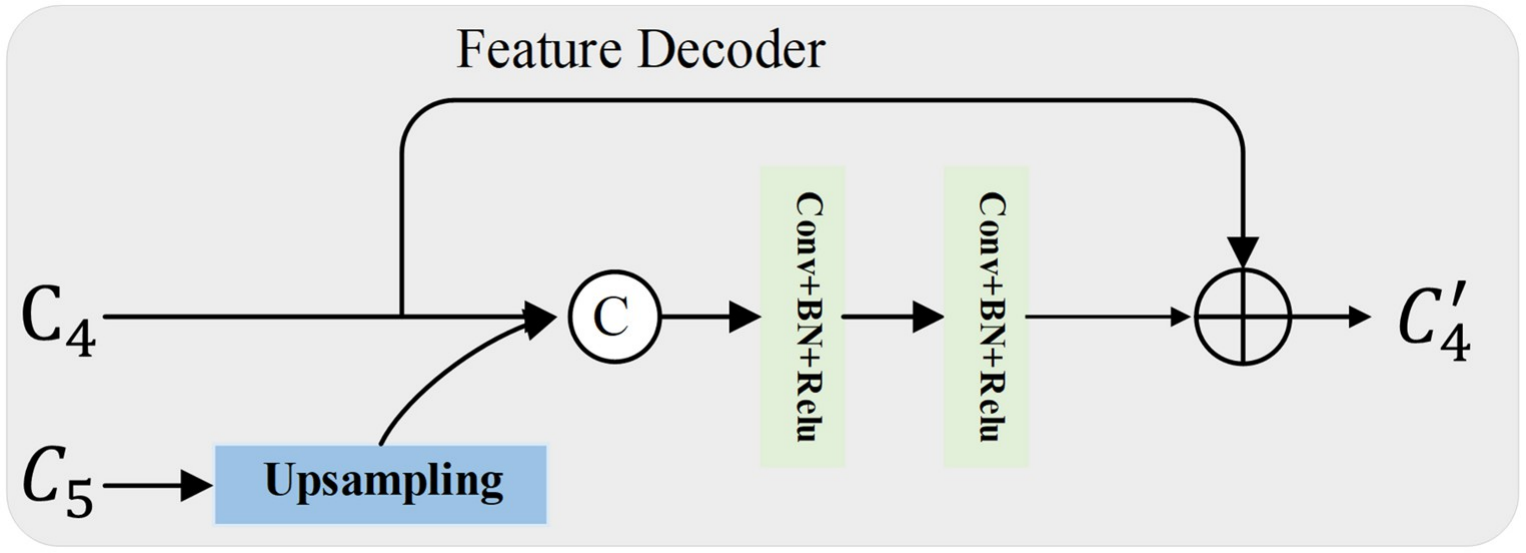
The structural design of the feature decoder, exemplified here by *C*_4_ and *C*_5_.

It is observed that foreground text instances occupy only a very small region in the image, resulting in relatively sparse useful information for locating the text position. Additionally, the extreme distribution of scene text scale and aspect ratio necessitates sufficiently rich detail information to make accurate judgments in scene text detection tasks. Aiming at the above problems, this paper optimizes the Feature Pyramid Network (FPN) and proposes a decoder with a feature sampling strategy. This approach enhances and supplements the text feature detail information, providing finer features. Consequently, this improves the feature learning ability of the model and enhances its stability. The feature decoder is primarily constructed using the traditional convolution mechanism, as illustrated in Fig 3. In this paper, the feature decoder first processes the output features from the feature encoder through a standard convolution layer, reducing the number of channels to 128. Subsequently, upsampling is performed to double the size of the feature map. A concatenation operation is performed with the upper layer (*C*_4_). Then, a skip connection is made between the output result and the feature map *C*_4_, which is output by the original ResNet. The skip connection supplies initial features to the feature decoder, enabling access to the local features of the high-resolution feature map from the feature encoder. This enhances feature delivery and bridges the semantic gap between the encoder and decoder. Finally, the number of channels is restored to 128 through a standard convolution layer. Repeat the above operation for the output result, and continue with the concatenation operation with its upper layer (feature map *C*_3_). Its output is then connected via a skip connection to the feature map *C*_3_, which is output by the original ResNet. Finally, the number of channels is restored to 128 through a standard convolution layer. From the bottom up, this process is repeated three times until reaching the feature map *C*_2_. This repetition allows the feature decoder to access the local features of the high-resolution feature map from the encoder. Taking feature layers *C*_4_ and *C*_5_ as an example, this process is represented by the mathematical Eq (2):

$$C_{4}^{\prime }=\text{Conv}(\text{Concat}(\text{Up}(\text{Conv}(C_{5})),C_{4})+C_{4})$$ (2)

Where $C_{4}^{\prime }$ is the output result. Through the feature sampling strategy and the excellent skip connection, the features of the decoder are integrated, significantly balancing the relationship between computational complexity and the performance of the model. On the other hand, it can effectively integrate global, local, and multi-scale features to facilitate excellent feature extraction and enhance the ability of scene text detection.

### Differentiable binarization module

**Fig 4:**
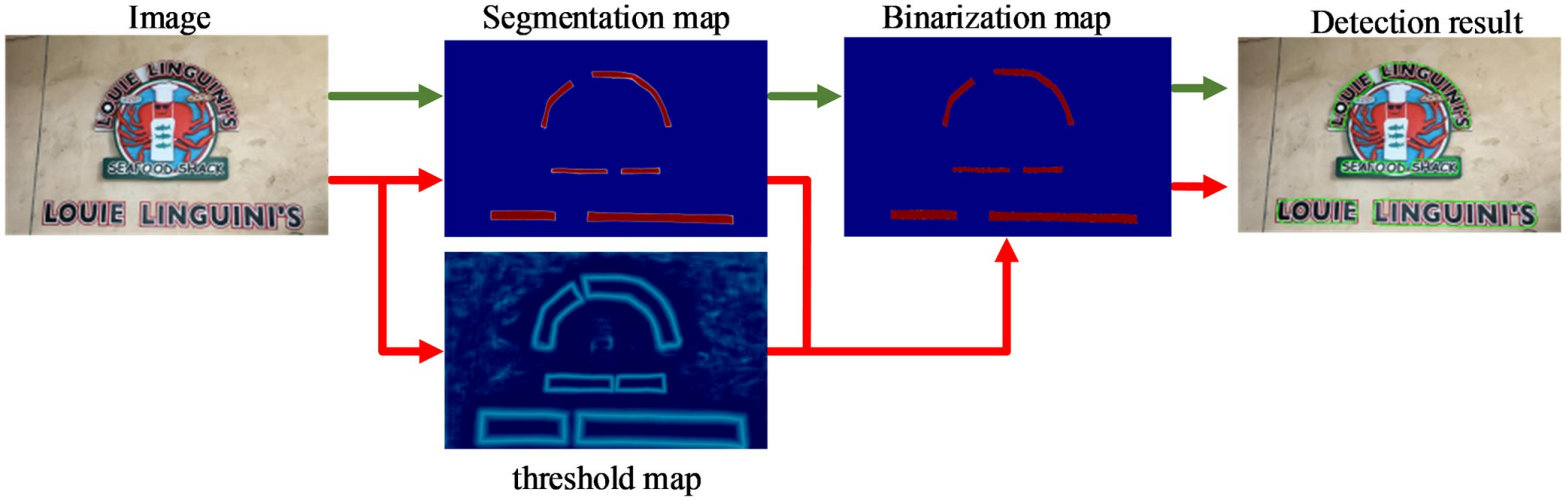
Traditional path (green flow) and our path (red flow). The green arrows represent the standard binarization process. The red arrows represent differentiable binarization, the method adopted in this paper, which can adaptively predict the threshold for each position in the image.

Fig 4 illustrates the differentiable binarization structure, which generates the probability map (P) and the threshold map (T) from features. The binarization module combines the probability map and the threshold map to produce a binarization map, which facilitates adaptive prediction of the threshold value for each position. Finally, the detection box, used to extract text from the approximate binary map, is derived from the bounding box. Red arrows denote differentiable binarization, while green arrows signify standard binarization. The standard binarization function is presented in Eq (3), where a value of 1 indicates an effective text area.


$$B_{i,j}=\left\{\begin{array}{ll} 1, & P_{i,j}\geq t\\ 0, & otherwise \end{array}\right.$$ (3)

To solve the problem that Eq (3) is not differentiable, Eq (4) is utilized for binarization:

$$B_{i,j}^{\prime }=\frac{1}{1+e^{-k(P_{i,j}-T_{i,j})}}$$ (4)

*B*′ represents the approximate binary map, T denotes the adaptive threshold map, and K is the amplification coefficient. Consistent with baseline settings, *k* is set to 50.

### Loss function

The loss function remains consistent with the baseline. The loss function [2] used in this study is presented in Eq (5):

$$L=L_{S}+\alpha \times L_{b}+\beta \times L_{t}$$ (5)

where the weight parameters *α* are set to 1 and *β* to 10. *L*_*S*_ represents the probability map loss, *L*_*b*_ denotes the binarization map loss, and *L*_*t*_ signifies the adaptive threshold map loss. *L*_*s*_ and *L*_*b*_ both employ binary cross-entropy loss functions, as detailed in Eq (6):

$$L_{s}=L_{b}=\sum_{i\in S_{l}}\;y_{i}logx_{i}+\left(1-y_{i}\right)\text{log}\left(1-x_{i}\right)$$ (6)

*L*_*t*_ adopts the L1 loss function, as detailed in Eq (7):

$$L_{t}=\sum_{i\in R_{d}}\left|y_{i}^{*}-x_{i}^{*}\right|$$ (7)

where *R*_*d*_ represents the pixel index, and y* denotes the label of the adaptive threshold map.

## Experiments and results

**Fig 5:**
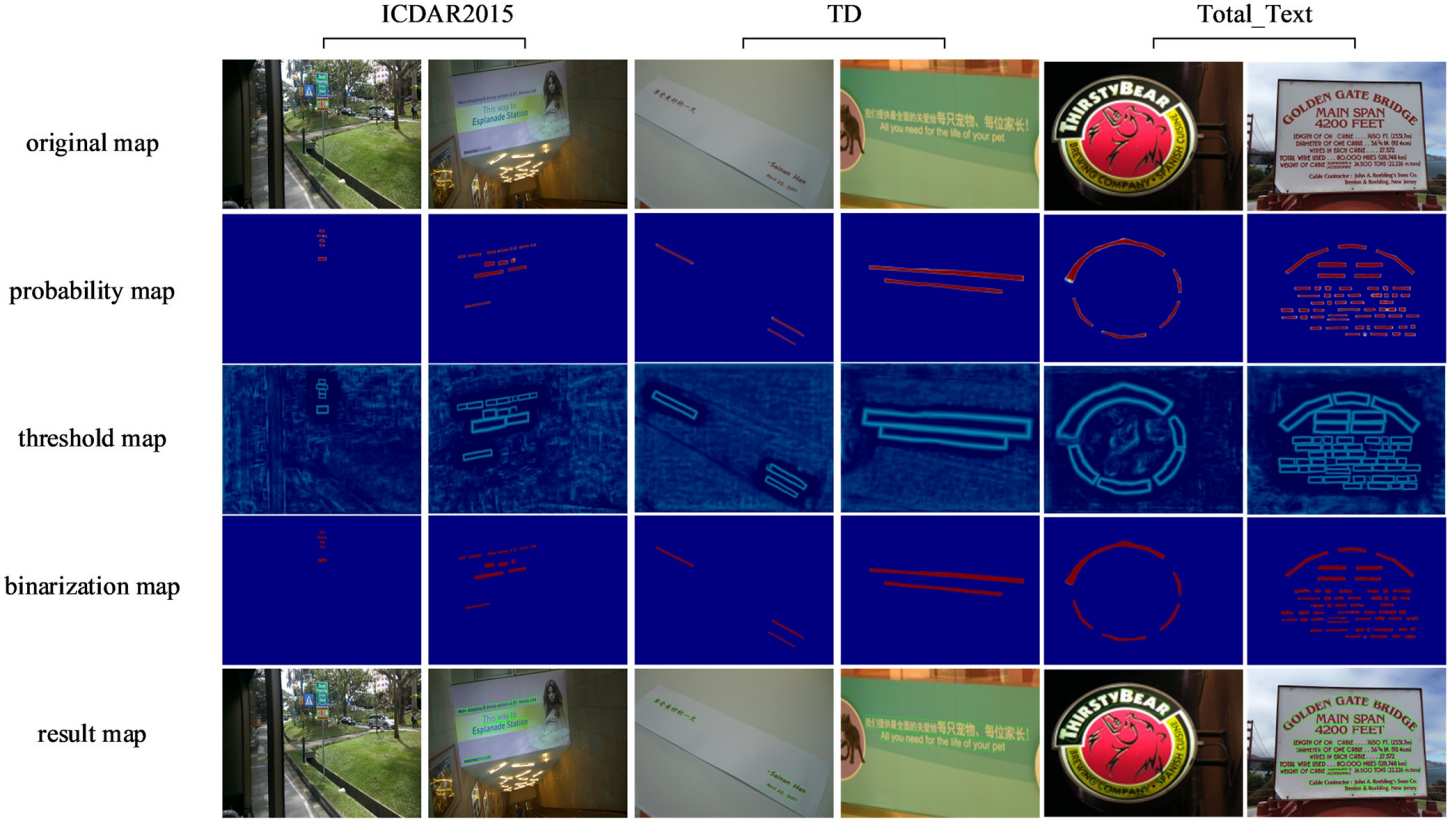
Visualization of the results of our method on different types of text instances, including multi-oriented, multi-lingual, and curved text. The second to fourth rows correspond to the probability map, the threshold map, and the binarization map for each text instance in the images, respectively.

Fig 5 displays the visual results of the model when tested on three different types of datasets; the middle three rows correspond to the probability map, the threshold map, and the binarization map, respectively.

ICDAR2015 contains a total of 1500 images, where the ratio of training set to test set is 2:1 and the image resolution is 1280×720. This is a multi-orientational street-view dataset primarily used for detecting text in arbitrary orientations. Text regions are annotated at the word level using the four vertices of a quadrilateral. This dataset primarily consists of English text and numbers. The text size within the images is inconsistent and exhibits significant variations. It is widely used as a benchmark dataset for text detection.

MSRA-TD500 has a total of 500 multi-category and multi-language images, which contains 300 training images and 200 test images. The dataset focuses on multilingual quadrilingual text in natural scenes, including English and Chinese. The resolution of the images ranges from 1296×864 to 1920×1280. The dataset has a larger aspect ratio compared to the other datasets and contains mainly photographs taken in natural scenes such as schools, stores and streets.

Total-Text contains a total of 1555 images, of which 1255 are training images and 300 are test images. These images are from different real-world scenarios, including some text-like complex backgrounds and low-contrast text. In addition, these images contain text in more than three different orientations (horizontal, multi-directional and curved) and are labeled with polygons at the word level for curved text detection.

The performance evaluation of the text detection algorithm is determined by precision (P), recall (R), and the F-measure [2]. Precision refers to the accurate detection of text targets. Recall refers to the text targets that should be detected. The composite metrics is the result of a weighted harmonic mean, used to evaluate the overall performance of the algorithm. Among these metrics, the F-measure is the most important, being related to both precision and recall. It can be considered that a higher F-measure indicates better detection performance of the model. The calculations for these three metrics are given in Eqs (8), (9) and (10):

$$P=\frac{TP}{TP+FP}$$ (8)

$$R=\frac{TP}{TP+FN}$$ (9)

$$F=2\times \frac{P\times R}{P+R}$$ (10)

Herein, TP (True Positive) represents the text areas that have been correctly detected, FP (False Positive) represents the background areas mistakenly identified as text areas, and FN (False Negative) denotes the text areas that have been missed.

### Implementation details

The experiments were conducted using the PyTorch 1.7.1 deep learning framework. All training was conducted on an NVIDIA RTX A4000 GPU, with the images sized at 640x640. In this paper, the Adam optimizer is employed as the learning strategy for network optimization, with an initial learning rate set at 0.001 and a training batch size of 16. In all three datasets, all models were trained and tested under uniform strategy and settings, respectively. In this paper, DBNet was reproduced in a consistent environment to ensure the validity of the comparative analysis.

### Experiments

#### Ablation experiment

This subsection discusses the effectiveness of the module proposed in this paper, validated from the following perspectives:

1. with and without CESAM and SESAM.
2. with and without featured decoders.
3. number of attention heads and depth.

Evaluation of performance includes precision (P), recall (R), and composite metrics (F-score). The experiments were conducted in a consistent environment, and the results are presented in Tables 2 and 3.

**Table 3:** Test results in MSRA-TD500 dataset.

model	box_size	P(%)	R(%)	F(%)
ResNet18+FPN+DB(Baseline)	736	86.04	77.32	81.45
ResNet18+ CESAM +FPN+DB	736	86.96	80.24	83.47
ResNet18+ SESAM +FPN+DB	736	87.76	78.87	83.08
ResNet18+ dual +FPN+DB	736	88.05	82.33	85.1
ResNet18+dual+decoder+DB	736	88.87	82.3	**85.46**

A series of ablation experiments were conducted on the MSRA-TD500 dataset to validate the effectiveness of CESAM, SESAM, and the feature decoder. Table 3 evaluates whether the CESAM, SESAM, and feature decoder proposed in this paper enhance the model’s detection capabilities. As observed on the MSRA-TD500 dataset, the model incorporating CESAM demonstrates increases of 0.92% in precision (P), 2.92% in recall (R), and 2.02% in F-score compared to the baseline. The CESAM proposed in this paper demonstrates a significant advantage over traditional CNNs by effectively perceiving the global information of the input sequence. The introduction of SESAM in the model resulted in increases of 1.72% in precision (P), 1.55% in recall (R), and 1.63% in F-score compared to the baseline. The ‘dual’ denotes the incorporation of both CESAM and SESAM, which results in increases of 2.01% in precision (P), 5.01% in recall (R), and 3.65% in F-score compared to the baseline. It is evident that the detection performance from integrating two modules significantly surpasses that achieved by incorporating only one module. Finally, with the integration of CESAM, SESAM, and a feature decoder, the ResNet18+dual+decoder+DB (ours) achieved an accuracy of 88.87%, a recall of 82.3%, and an F-score of 85.46%. Compared to the baseline, precision (P) increased by 2.83%, recall (R) by 4.98%, and F-score by 4.01%. The results indicate that ResNet18+dual+decoder+DB outperforms ResNet18+FPN+DB (DBNet), ResNet18+CESAM+FPN+D*B*, ResNet18+SESAM+FPN+D*B*, and ResNet18+dual+FPN+D*B*, further proving the effectiveness of the proposed CESAM, SESAM, and feature decoder. Therefore, the model proposed in this paper effectively extracts features, ensuring the completeness of text feature information to a considerable extent. This enhancement significantly improves both the feature learning capability and the image detection ability of the model.

The comparative results of network parameters, computational complexity (FLOPs), and detection performance are presented in Table 4. As evidenced by Table 4, when the number of attention heads is set to 4 and the depth to 2, the network parameters, FLOPs, and detection performance of the model presented in this study achieve optimal results. When the depth is set to 2, using 4 attention heads as opposed to 6 or 8 results in a reduction of parameter count by 10% (14.14 vs. 14.24), and computational complexity (FLOPs) by 5% (38.7 vs. 38.75) and 9% (38.7 vs. 38.79), respectively. The model also demonstrated enhanced performance: with 4 attention heads compared to 8, precision (P) increased by 0.33% and 0.02%, F-measure (F) by 0.15% and 0.12%, and recall (R) by 0.21%. It has been observed that, with constant depth, increasing the number of attention heads does not necessarily improve network parameters, computational complexity (Flops), or detection performance. Similarly, when the number of attention heads remains fixed, the influence of varying depth levels should also be systematically examined. As depth increases, the network parameters and computational complexity (Flops) correspondingly increase. Conversely, performance metrics indicate that although precision (P) improves marginally with increased depth, both recall (R) and the F-score (F) tend to decrease. Consequently, this study adopts a model configuration with 4 attention heads and a depth of 2 as the optimal architecture.

**Table 4:** Parameter settings and performance comparison on feature encoder.

model	num_heads	depth	Parameters (M)	Flops (G)	box_size	P	R	F
ResNet18+dual+decoder+DB	4	2	14.14	38.7	736	88.87	82.3	**85.46**
	6	2	14.24	38.75	736	88.54	82.3	85.31
	8	2	14.24	38.79	736	88.85	82.09	85.34
	4	4	14.61	38.9	736	89.22	81.7	85.3
	4	5	14.84	39	736	90.6	80.79	85.42
	4	6	15.07	39.09	736	90.93	80.6	**85.45**

**Fig 6:**
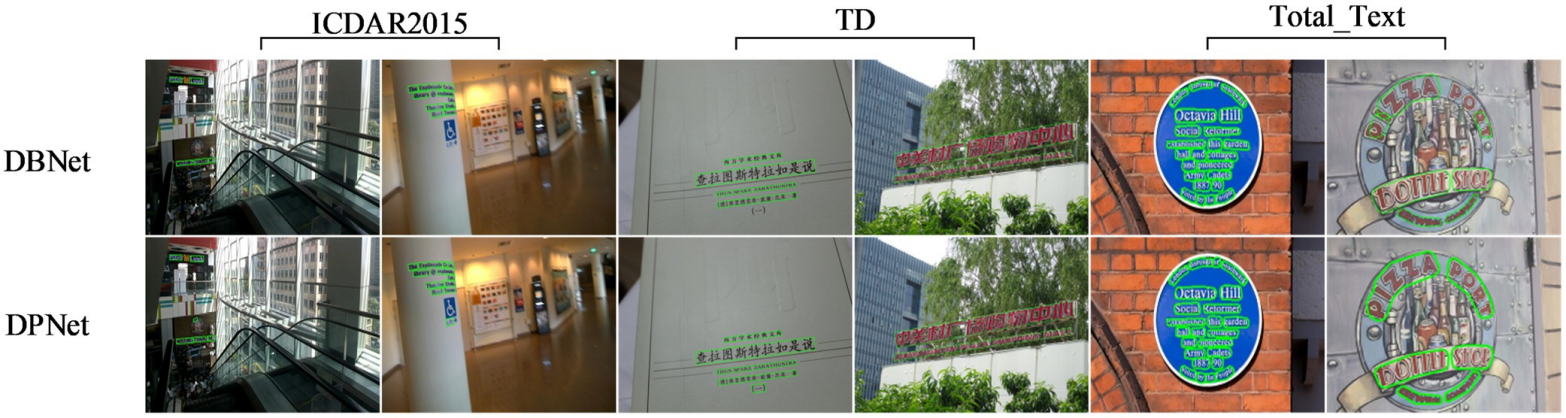
Comparison of visualization results for baseline and DPNet. Under the combined effect of the proposed CESAM, SESAM, and feature decoder, DPNet achieves more precise text box positioning, effectively addressing the issues of missed and false detections, proving to be more effective compared to the Baseline.

Fig 6 presents a visualization comparison between the Baseline model and DPNet. In the ICDAR 2015 dataset, the Baseline model tends to overlook areas with relatively small text fonts and misaligned text, while DPNet demonstrates improved attention to these areas. In the MSRA-TD500 dataset, the Baseline model exhibits limited sensitivity to text feature information, often failing to detect certain characters, such as ‘(一)’ depicted in the figure. In contrast, DPNet demonstrates accurate detection of these elements. Furthermore, the Baseline model lacks a profound understanding of the semantic information of text, which can easily lead to biases and impact the precision of text box representation. In comparison, DPNet achieves superior results, yielding more accurate text boxes. In the Total-Text dataset, DPNet demonstrates a high detection rate for curved and arbitrary-shaped texts, achieving both zero omissions and zero misdetections. The experimental results demonstrate that DPNet effectively enhances the focus on text features and utilizes these features efficiently, which plays a crucial role in the training of the model.

#### Comparison with other algorithms

To validate the efficacy of DPNet, the proposed method was compared with previous approaches on various datasets, including the multi-directional text dataset ICDAR2015, the curved text dataset Total-Text, and the multilingual text dataset MSRA-TD500. The network demonstrates strong performance under natural scene text detection datasets, exhibiting high precision, recall, and F-measure, resulting in a more efficient network.

After extensive training, the DPNet proposed in this paper is benchmarked against established methods on the Total-Text dataset, as shown in Table 5. These methods include TextSnake [39], ATRR [43], TextField [44], LOMO [45], CRAFT [42], PSE [40], Mask-TTD [46], TFE-PRPA-BCTS [47], DiffBiSeg [48, 49], KPN [50], RP-Text [51] and Buffer-Text (FCN) [52]. The P, R, and F values for DPNet-ResNet18 (800×800) are 85.87%, 78.81%, and 82.19%, respectively. This represents an improvement of 4.61% in R and 2.04% in F compared to DBNet-18 (800×800). The P, R, and F values for DPNet-ResNet50 (800×800) are 87.56%, 80.37%, and 83.81%, respectively. These values represent improvements of 3.26%, 1.97%, and 2.51% in P, R, and F, respectively, compared to DBNet-18 (800×800). The results demonstrate significant improvements and even outperform existing scene text detection algorithms. DPNet-ResNet50 (800×800) achieves the highest recall (R), indicating that more text is detected, which enhances the precision of the detection area and benefits subsequent text recognition tasks. Therefore, the results of the above experiments demonstrate that the model proposed in this paper effectively delineates finer boundary regions, enhances detection capabilities, and adapts well to curved texts of arbitrary shapes.

**Table 5:** Test results for the total-text dataset (values in parentheses refer to the height of the input image).

Method	Parameters (M)	Flops (G)	P(%)	R(%)	F(%)
TextSnake (2018)	19.1	136.01	82.7	74.5	78.4
ATRR (2019)	-	-	80.9	76.2	78.5
TextField (2019)	-	-	81.2	79.9	80.6
LOMO (2019)	-	-	87.6	79.3	83.3
CRAFT (2019)	20.8	146.29	87.6	79.9	83.6
PSENet (2019)	28.6	117.1	84.0	78.0	80.9
Mask-TTD (2020)	-	-	79.1	75.4	76.7
TFE-PRPA-BCTS (2020)	-	-	84.6	78.6	81.5
DiffBiSeg (2021)	-	-	88.4	77.9	82.8
[49] (2023)	-	-	85.8	77.0	81.1
KPN (2023)	-	-	88.0	82.33	85.07
RP-Text (2023)	-	-	89.4	82.8	86.0
Buffer-Text (FCN) (2024)	-	-	76.9	77.5	77.2
**DBNet-18 (800×800)**	12.18	26.29	87.13	74.2	80.15
**DPNet-ResNet18 (800×800)**	14.14	38.7	85.87	78.81	82.19
**DBNet-50 (800×800)**	25.25	47.49	84.3	78.4	81.3
**DPNet-ResNet50 (800×800)**	26.84	58.72	87.56	80.37	**83.81**

In addition, the parameters and Flops of the Baseline, DPNet, and other models are presented in Table 5. It can be seen that, compared with the Baseline, DPNet-ResNet18 (800×800) introduces a slight increase in parameters and Flops, while its performance improves by 2.04% (82.19 vs. 80.15). The parameters and complexity of our ResNet50 (800×800) model have increased slightly, yet the performance has improved by 2.51%. Compared with PSE-1, this model not only achieves better performance but also requires fewer parameters and lower Flops. In summary, the model proposed in this paper utilizes fewer parameters and Flops, while effectively retaining highly representative features for detection. Additionally, it leverages a unique feature aggregation capability to accurately determine detection boxes without the need for any post-processing.

After sufficient training, the DPNet proposed in this paper was compared with advanced methods in ICDAR 2015, as detailed in Table 6. These methods include EAST [38], Corner [53], RRD [54], PAN [41], PSE [40], SRPN [55], CFPM-IDB [56], DiffBiSeg [48], PCBSNet [49, 57], KPN [50], RP-Text [51], and RetinaDB [58]. The precision (P), recall (R), and F-score (F) of DPNet-ResNet18 (1280×736) are 88.58%, 77.03%, and 82.41%, respectively, representing improvements of 2.34%, 1.87%, and 2.09% over DBNet-18 (1280×736). Compared with EAST, the P, R and F of DPNet-ResNet18 (1280×736) improved by 4.98%, 3.53% and 4.21%. The P, R and F of DPNet-ResNet50 (1280×736) are 90.23%,79.15% and 84.33% respectively, showing improvements of 0.59%, 3.37%, and 2.2% over DBNet-50 (1280×736) in these metrics. The P, R and F of DPNet-ResNet50 (2048×1152) are 91.91%,80.93% and 86.07%, respectively, registering improvements of 2.11% in precision, 1.63% in recall, and 1.87% in F-score compared to DBNet-50 (2048×1152). The experimental results demonstrate that compared to the baseline, DPNet significantly enhances the boundary localization capabilities of text instances by fully exploiting rich feature information, thanks to its efficient fusion mechanism and functionality enhancements. Moreover, with an F-score of 86.07%, DPNet surpasses hybrid algorithms such as EAST, Corner, and SRPN, achieving state-of-the-art performance.

**Table 6:** Test results for the ICDAR 2015 dataset (values in parentheses indicate the height of the input image).

Method	P(%)	R(%)	F(%)
EAST (2017)	83.6	73.5	78.2
Corner (2018)	94.1	70.7	80.7
RRD (2018)	85.6	79.0	82.2
PAN (2019)	84.0	81.9	82.9
PSE (2019)	86.9	84.5	85.7
SRPN (2020)	92.0	79.7	85.4
CFPM-IDB (2020)	88.6	81.4	84.8
DiffBiSeg (2021)	86.9	78.6	82.5
PCBSNet (2023)	88.0	78.4	82.9
[49] (2023)	88.1	78.8	83.2
KPN (2023)	84.08	83.15	83.61
RP-Text (2023)	89.6	82.4	85.9
RetinaDB (2024)	83.91	78.83	81.29
**DBNet-18 (1280×736)**	86.24	75.16	80.32
**DPNet-ResNet18 (1280×736)**	88.58	77.03	82.41
**DBNet-50 (1280×736)**	89.64	75.78	82.13
**DPNet-ResNet50 (1280×736)**	90.23	79.15	84.33
**DBNet-50 (2048×1152)**	89.8	79.3	84.2
**DPNet-ResNet50 (2048×1152)**	91.91	80.93	**86.07**

After sufficient training, the DPNet proposed in this paper is compared with advanced methods in MSRA-TD500 (see Table 7), including EAST [38], DeepReg [59], RRPN [60], RRD [54], PixelLink [61], Corner [53], TextSnake [39], CRAFT [42], SAE [62], PAN [41], SRPN [55], DiffBiSeg [48], AS-RPN [63], RelaText [64], PCBSNet [49, 57], SPW [65] and RP-Text [51]. The P, R, and F values for DPNet-ResNet18 (512×512) are 90.48%, 75.09%, and 82.07%, respectively, representing increases of 3.72%, 4.13%, and 4.00% in P, R, and F compared to DBNet-18 (512×512). The P, R, and F values of DPNet-ResNet18 (736×736) are 88.87%, 82.3%, and 85.46%, respectively, showing improvements of 2.83%, 4.98%, and 4.01% in P, R, and F compared to DBNet-18 (736×736). The P, R, and F values of DPNet-ResNet50 (736×736) are 91.43%, 82.47%, and 86.72%, respectively, representing increases of 3.21%, 3.95%, and 3.63% in P, R, and F when compared with DBNet-18 (736×736). The experimental results demonstrate that DPNet-ResNet50 (736×736) delivers the best performance among the algorithms evaluated, achieving the highest F-score. The detection results significantly surpass those of the original DBNet, proving that our model is robust for multilingual text detection and is practically applicable in complex natural scenes. Compared to the state-of-the-art algorithm RP-Text, our approach achieved a higher precision (86.72 vs. 86.5).

**Table 7:** Test results on MSRA-TD500 dataset (values in parentheses are heights of input images).

Method	P (%)	R (%)	F (%)
EAST (2017)	87.28	67.43	76.08
DeepReg (2017)	77.0	70.0	74.0
RRPN (2018)	82.0	68.0	74.0
RRD (2018)	87.0	73.0	79.0
PixelLink (2018)	83.0	73.2	77.8
Corner (2018)	87.6	76.2	81.5
TextSnake (2018)	83.2	73.9	78.3
CRAFT (2019)	88.2	78.2	82.9
SAE (2019)	84.2	81.7	82.9
PAN (2019)	84.4	83.8	84.1
SRPN (2020)	84.9	77.0	80.7
DiffBiSeg (2021)	85.9	73.2	79.0
AS-RPN (2021)	84.7	80.4	82.5
RelaText (2021)	90.5	83.2	86.7
PCBSNet (2023)	90.0	76.7	82.8
[49] (2023)	90.0	80.4	84.9
SPW (2023)	89.8	83.1	86.3
RP-Text (2023)	88.4	84.6	86.5
**DBNet-18 (512×512)**	86.76	70.96	78.07
**DPNet-ResNet18 (512×512)**	90.48	75.09	82.07
**DBNet-18 (736×736)**	86.04	77.32	81.45
**DPNet-ResNet18 (736×736)**	88.87	82.3	85.46
**DBNet-50 (736×736)**	88.22	78.52	83.09
**DPNet-ResNet50 (736×736)**	91.43	82.47	**86.72**

**Fig 7:**
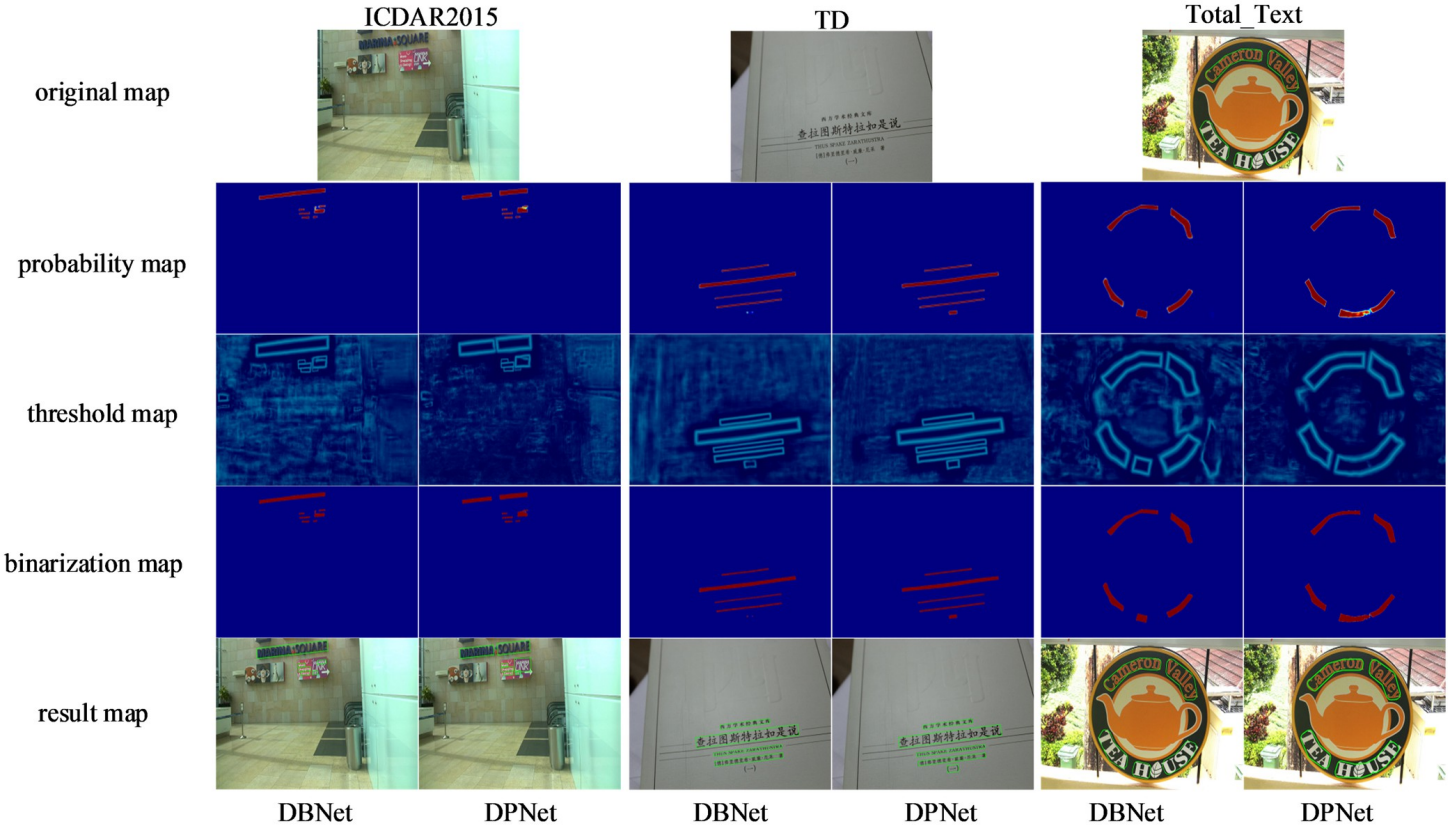
Visualization results of text instances for our method DPNet and the baseline on different types of datasets. The images are randomly selected from three datasets, which better demonstrate the robustness of our model.

The visualization results of DPNet and Baseline on different types of text samples are illustrated in Fig 7. The middle three rows correspond to the probability map, the threshold map, and the binarization map, respectively; the last row presents the final detection result. The visualization results presented in the paper depict images randomly selected from three datasets, further demonstrating the robustness of the model. In the ICDAR 2015 dataset, it was observed that the Baseline model missed portions of text in the images, specifically the ‘MA’ in ‘LINK’ and ‘MARINA’, which were successfully detected by DPNet. Furthermore, DPNet demonstrates greater accuracy in comprehending the semantic information of the text ‘MARINA SQUARE’. In the MSRA-TD500 dataset, while the Baseline model failed to detect the text ‘(一)’, DPNet successfully detected it. In the curved text dataset Total-Text, due to the effects of the background and special characters, the Baseline model erroneously detects "HUSE" as two separate textual components, overlooking the semantic meaning represented by the word itself. In reality, the leaf also represents a character, and when combined with "HUSE," it conveys the semantic information of "HOUSE." Therefore, it should be detected as a single textual region. In terms of visualization, it can be observed that the positioning information of text boxes by DPNet is more accurate compared to DBNet, further demonstrating that our method (DPNet) is more effective than the baseline (DBNet).

## Discussion

It is evident that the network proposed in this paper outperforms the Baseline model across multiple scene text detection datasets. In the feature extraction stage, the convolution kernel of the ResNet backbone network typically operates at a fixed size, such as 3×3, which results in low efficiency in capturing long-range dependencies. The CESAM and SESAM proposed in this paper effectively learn the interaction of information between text instances, capture contextual information over long distances, and demonstrate robust feature extraction capabilities. At the same time, this paper also proposes a well-designed feature decoder that enriches the capability of the original FPN structure in extracting feature information, thereby improving the performance of text detection. By incorporating the CESAM, SESAM, and feature decoder proposed in this paper into the Baseline model, a more efficient model can be achieved. This integration demonstrates the effectiveness and significant contributions to the field of scene text detection.

Based on the ablation studies and comparative experiments presented above, it has been discovered that the algorithm proposed in this paper significantly improves performance compared to the baseline. Additionally, there is still room for further optimization of its parameter count and complexity in future work. In future work, this paper will continue to explore the lightweight design of the model, aiming to simultaneously optimize network parameters, computational complexity, and network performance. The goal is to develop a more efficient text detection network framework that offers enhanced real-time capabilities. For instance, one could consider applying a Fourier transform to the features, followed by learning a content-adaptive filtering mask in the frequency domain. This would retain only the textual feature information, thereby achieving a lightweight and efficient model. Furthermore, subsequent research will continue to explore the text detection capabilities of the model in specific weather conditions, such as rainy, foggy, and snowy scenarios, to ensure effective performance under various meteorological conditions.

## Conclusions

This paper proposes a scene text detection algorithm based on a dual-perspective CNN-transformer, built upon the foundation of DBNet. To address the deficiencies in feature extraction capabilities, this paper proposes CESAM, SESAM, and a feature decoder to enhance the feature extraction process. Firstly, the CESAM and SESAM proposed in this paper are integrated into the backbone network to construct powerful global contextual information and enhance feature extraction capabilities. Secondly, the feature decoder proposed in this paper is designed to enhance the representation of feature information for arbitrary text, thereby significantly improving detection performance. This study primarily addresses the detection of small and arbitrarily shaped texts. The experimental and visualization results confirm the efficacy of the methods proposed. However, challenges such as occlusions and color distortions remain areas for further exploration. In conclusion, DPNet no longer relies purely on CNNs for detection, which effectively reduces instances of missed and false detections. In future work, DPNet will be further improved and optimized. By combining traditional methods with deep learning approaches, a more efficient architecture will be designed to better balance performance and applicability. Additionally, it should possess enhanced generalization capabilities, enabling its application in a wider range of natural environments.
